# Spatial distribution and habitat characterization of mosquito species during the dry season along the Mara River and its tributaries, in Kenya and Tanzania

**DOI:** 10.1186/s40249-017-0385-0

**Published:** 2018-01-18

**Authors:** Gabriel O. Dida, Douglas N. Anyona, Paul O. Abuom, Daniel Akoko, Samson O. Adoka, Ally-Said Matano, Philip O. Owuor, Collins Ouma

**Affiliations:** 1 School of Public Health and Community Development, Maseno University, Maseno, Kenya; 2 School of Environment and Earth Sciences, Maseno University, Maseno, Kenya; 3 Impact Research and Development Organization, Kisumu, Kenya; 4 School of Health Sciences, Jaramogi Oginga Odinga University of Science and Technology, Bondo, Kenya; 5 East African Community/Lake Victoria Basin Commission, Kisumu, Kenya; 6 Department of Chemistry, Maseno University, Maseno, Kenya; 7 Department of Community and Public Health, Technical University of Kenya, Nairobi, Kenya

**Keywords:** *Anopheles gambiae*, *Anopheles funestus*, *Culex*, Mosquito, Larval habitat, Mara River, Kenya, Tanzania

## Abstract

**Background:**

Vector-borne diseases are increasingly becoming a major health problem among communities living along the major rivers of Africa. Although larger water bodies such as lakes and dams have been extensively researched, rivers and their tributaries have largely been ignored. This study sought to establish the spatial distribution of mosquito species during the dry season and further characterize their habitats along the Mara River and its tributaries.

**Methods:**

In this cross-sectional survey, mosquito larvae were sampled along the Mara River, its two perennial tributaries (Amala and Nyangores), drying streams, and adjacent aquatic habitats (e.g. swamps, puddles that receive direct sunlight [open sunlit puddles], rock pools, hippo and livestock hoof prints, and vegetated pools). Each habitat was dipped 20 times using a standard dipper. Distance between breeding sites and human habitation was determined using global positioning system coordinates. The collected mosquito larvae were identified using standard taxonomic keys. Water physico-chemical parameters were measured in situ using a multiparameter meter*.* Mean mosquito larvae per habitat type were compared using analysis of variance and chi-square tests, while the relationship between mosquito larvae and physico-chemical parameters was evaluated using a generalized linear mixed model. The Cox-Stuart test was used to detect trends of mosquito larvae distribution. The test allowed for verification of monotonic tendency (rejection of null hypothesis of trend absence) and its variability.

**Results:**

A total of 4001 mosquito larvae were collected, of which 2712 (67.8%) were collected from river/stream edge habitats and 1289 (32.2%) were sampled from aquatic habitats located in the terrestrial ecosystem about 50 m away from the main river/streams. *Anopheles gambiae s.s*, *An. arabiensis*, and *An. funestus* group, the three most potent vectors of malaria in Sub-Saharan Africa, together with other anopheline mosquitoes, were the most dominant mosquito species (70.3%), followed by *Culex quinquefasciatus* and *Cx. pipiens* complex combined (29.5%). Drying streams accounted for the highest number of larvae captured compared to the other habitat types. A stronger relationship between mosquito larvae abundance and dissolved oxygen (Z = 7.37, *P* ≤ 0.001), temperature (Z = 7.65, *P* ≤ 0.001), turbidity (Z = −5.25, *P* ≤ 0.001), and distance to the nearest human habitation (Z = 4.57, *P* ≤ 0.001), was observed.

**Conclusions:**

Presence of malaria and non-malaria mosquito larvae within the Mara River basin calls for immediate action to curtail the insurgence of vector-borne diseases within the basin. A vector control program should be conducted during the dry period, targeting drying streams shown to produce the highest number of larval mosquitoes.

**Electronic supplementary material:**

The online version of this article (10.1186/s40249-017-0385-0) contains supplementary material, which is available to authorized users.

## Multilingual abstracts

Please see Additional file 1 for translation of the abstract into the six official working languages of the United Nations.

## Background

Malaria and other infectious diseases are common in most tropical and sub-tropical regions. In 2015, it was estimated that 212 million cases of malaria occurred worldwide, with Sub-Saharan Africa bearing the greatest burden [1].

In Kenya, malaria accounts for 30% of outpatient attendance and 19% of hospital admissions [2]. A six-year surveillance study conducted across Kenya, from 2003 to 2009, indicated that of the 166 632 pediatric admissions, Western Kenya reported the highest number of malaria cases (70%), followed by the highland areas of Rift Valley (45%) and the Kenyan coast (22%) [2].

Due to the effects of climate change, important African lakes and rivers have receded incredibly, creating suitable breeding habitats for mosquitoes. This has thus resulted in increased malaria cases and other vector-borne diseases [3–6]. Climate change has led to disease outbreaks, especially in areas where such diseases were previously rare [7, 8], and the subsequent re-emergence of other disease transmitting pathogens such as those of microfilariae, arboviruses, and Chikungunya and O’nyong’nyong viruses [9, 10].

Mosquitoes of the genus *Anopheles* have been incriminated in the transmission of malaria in Kenya. Malaria epidemics occur frequently in the highlands, the Lake Victoria basin, and coastal regions. The following areas are cited as being most at risk: West Pokot, Trans-Nzoia, Uasin Gishu, Kericho, Nandi, Bureti, Kisii, Nyamira, Gucha, Transmara, and Nyando. Almost three-quarters of these fall within the Mara River basin catchment of Kenya [11].

In Tanzania, malaria is common in almost all regions, including the Maasai Mara National Reserve, which has been classified as a low to moderate malaria-epidemic area in East Africa [12]. According to the 2013 Serengeti Mara Camp Fact Sheet “http://static1.1.sqspcdn.com/static/f/648625/19311673/1342273530117/Fact+Sheet++Serengeti+Mara”, the famous Serengeti National Park in Tanzania falls within the malaria endemic zone.

The Mara River, which flows through the Maasai Mara National Reserve in Kenya and Serengeti National Park in Tanzania, has been impacted greatly by the wanton destruction of the Mau Forest, mainly on the upper catchment region [13, 14]. This has led to the reduction of Mara River water volume, and the subsequent alteration of water physico-chemical parameters and its hydrological characteristics [15]. However, it is not clear how and to what extent these changes have influenced the presence and distribution of mosquitoes within the basin. To evaluate this, it is necessary to establish which types of mosquito species breed within the Mara River basin. Studies conducted in Western Kenya have demonstrated that malaria larvae inhabit lagoons along the shores of Lake Victoria [6, 16] when the lake water recedes, especially during dry spells. More in-depth studies are, however, required to expand on these findings.

Larval habitats are crucial for determining the presence and density of different species of the vector, including the immature stages, as well as the abundance and distribution of adult mosquitoes. Understanding the dynamics and abundance patterns of mosquito larval habitats is therefore critical, if current efforts to model and understand adult mosquito distribution and abundance are to succeed. While considerable progress towards understanding the aquatic stages of mosquitoes has been made in Europe, Asia, and most parts of North America [17], substantial research on the larval ecology of the main malaria vectors in Africa is still limited [16, 18].

Most rivers and streams are often characterized by irrigation-supported agriculture along their continuum, some of which create aquatic microhabitats in the forms of stagnant waters, swamps, rock pools, and open sunlit puddles, which are ideal breeding sites for diverse mosquito species including vectors of malaria [10, 19]. However, information on the ecology of these potential vectors is limited or even unexplored in Kenya, especially in remote rivers and lakes around the country.

Thus, there was a need to establish the presence and distribution of mosquitoes along the Mara River and its basin to form a baseline model for other rivers in the country and beyond. The presence of malaria vectors along the Mara River and its tributaries, streams, and the adjacent habitats pose a health challenge to the local residents and tourists visiting the region, as they may have lower resistance compared to people who live in low altitude areas where exposure to malaria is normally high [20]. According to the 2010 Mara Travel Information Sheet “http://www.ieakenya.or.ke/number_of_the_week/number-of-visitors-to-national-parks-and-game-reserves-2011-2015”, each year 1–2 million tourists from various parts of the world visit the Maasai Mara National Reserve in Kenya and Serengeti National Park in Tanzania and stay in the area for several days; and that malaria is ranked as the first concern for travelers’ health in these areas.

Studies show that malaria re-emerged in the 1980s [21] owing to widespread mosquitoes in the highland regions of Kenya, as a result of destructive human activities coupled with climate change, which may create suitable habitats for mosquitoes to breed, as has been reported along Lake Victoria [16]. Such changes can also alter density and composition of mosquitoes in these habitats, as was observed in eastern Nepal [22]. However, without research, it is not possible to tell with certainty whether malaria-transmitting mosquitoes or those responsible for other diseases exist or not. Upon identification of the presence of different species and their distribution, their control becomes paramount. Historically, no information is available on malaria vector breeding habitats along the Mara River and adjacent aquatic ecosystems. Results from this study will therefore be valuable for malaria control initiatives in the context of effective vector management in rivers and adjacent habitats in the two East African countries, among others.

## Methods

### Study area

**Fig. 1:**
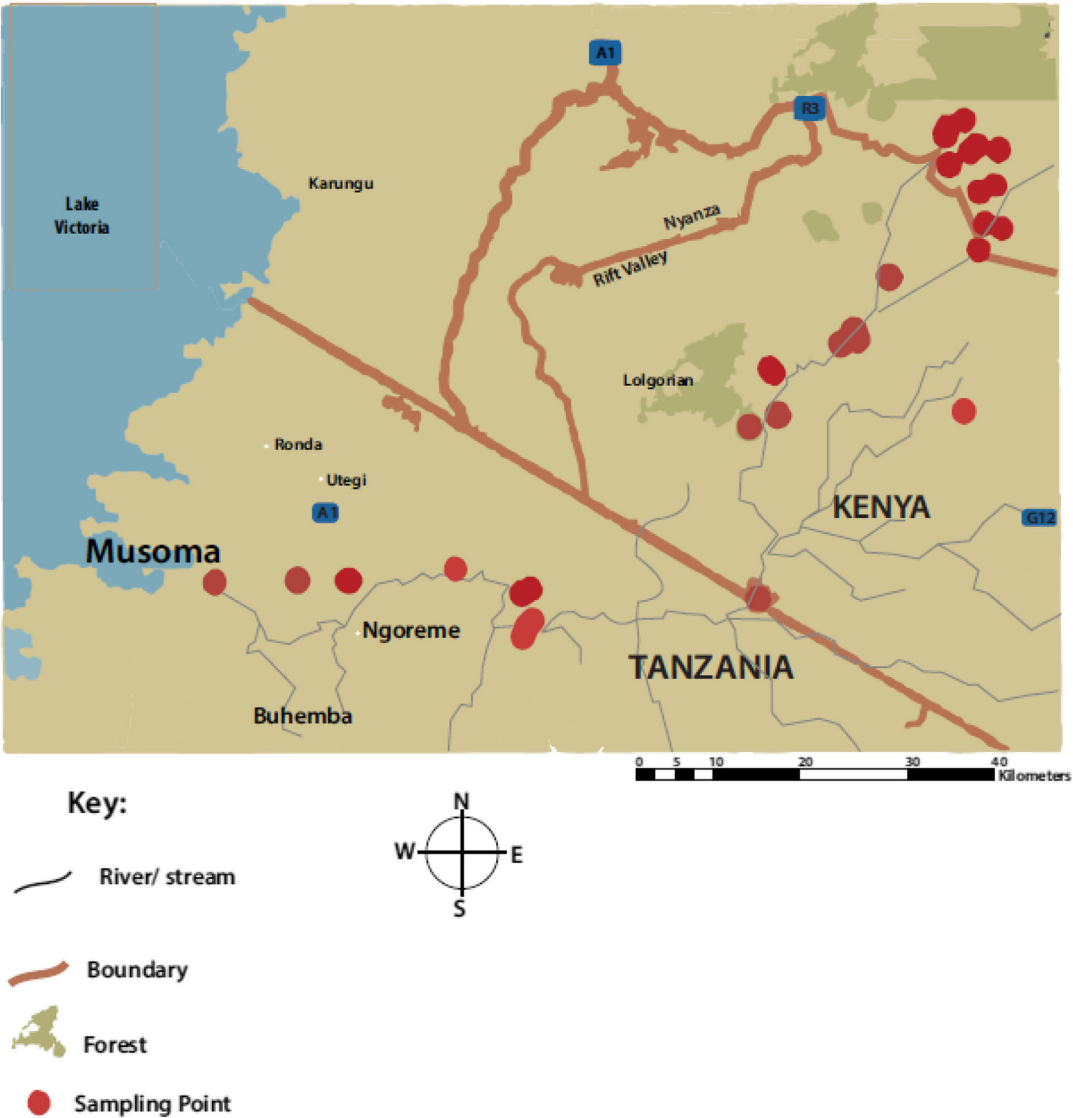
Map showing the study area (Kenya and Tanzania) and the geocoded location and distribution of the 39 sampling sites along the Mara River

This study was carried out along a transect within the Mara River basin located on the southwestern part of Kenya and the northeastern side of Tanzania. Sampling points were purposely selected by considering microhabitat type and accessibility along the Mara River, its two perennial tributaries (i.e. Amala and Nyangores), and several other feeder streams from first to fourth orders (Strahler system), which discharge their waters into the Amala and Nyangores tributaries (see Fig. 1).

The Mara River basin has bimodal rainfall seasons with long rains falling between mid-March and June, with peaks in April, and short rains between September and December. The dry season occurs between July and August, with a shorter period between December and February. The average precipitation is about 1400 mm annually, which varies between years, while evapotranspiration is around 1090 mm per year [13]. Given that different areas receive variable amounts of rainfall over the year, the drying streams are likely to be completely dry for an average of about 3 months in a year, while recording different volumes of water during the wet and short rain seasons depending on the amount of precipitation received. The changing water quantity also impacts on water quality characteristics of the different habitats.

The Mara-Serengeti ecosystem lies south of the equator and receives close to the maximum amount of the sun’s energy. Throughout the year, there is a constant mean monthly maximum temperature of 28 °C, with a mean minimum temperature range of between 16 °C in the hot months from October to March and 13 °C in the cooler months of May through to August, as according to the trans-boundary Mara River basin Strategic Environmental Assessment Report [23]. The upper Mara River catchment region has both large towns with populations of above 100 000, such as Mulot and Bomet, and smaller centers with populations of less than 100 000, such as Silibwet, Tendwet, Tenwek, Tegat, Kembu, and Mugango, among others. The lower Mara River catchment region lies in the northeastern part of Tanzania and has towns such as Kirumi, Serengeti, and Musoma.

The main economic activities in the area include large-scale livestock production and agriculture [14]. The mid-Mara region is characterized by a wildlife conservancy, which extends into the Maasai Mara National Reserve that meets the Serengeti National Park at the Kenya-Tanzania border. This region is characterized by wildlife with few hotels and tented camps. Lower Mara is predominantly covered by the Serengeti National Park and a gold mine, the activities of which are also thought to influence the water flow and velocity, as well as the water quality. Thereafter, the Mara River flows through small agropastoral lands, and finally discharges into Lake Victoria through the Nyansasura swamp at Kirumi in Musoma Bay, Tanzania.

### Study design

The sampling design included 39 main study sites along the Mara River and its tributaries (Amala and Nyangores) and several other smaller sites adjacent to them. The sampling points were purposively selected based on the presence of aquatic microhabitats and accessibility along the Mara River and its tributaries. Sampling was carried out from the beginning of July to the end of August 2011 (dry season) to evaluate the mosquito larvae bioburden and micro environmental extremes during the period of drought in the study area. The sampling sites were coded based on location and point of sampling. These points were at times strategically chosen before and after a bridge or a through-road (for ease of access to both sides of the river), and thus the sampling sites on either side of the bridge or road were labeled systematically. The first letters denoted their location as either being on the upstream or downstream part of the river, and the bridge was taken as the reference point. For example, URS 1–10 means that sampling sites 1 to 10 were located on the upper side of the main river or either of its tributaries before a bridge, while sampling points DRS 1–10 were located on the lower side of the river after the bridge. Other adjacent habitats were given Latin numbers with their nature and/or name of the habitat described in detail. This labeling system was employed to avoid confusion or mix-up of the results, and for easier analysis of the specimen.

### Mapping of mosquito breeding habitats

Raster images of the Mara River basin generated from Google Maps were used. Remote sensing techniques were used to classify and map features like water body, forest, vegetation cover, roads, and residential areas using the images generated from Google Maps. The data were digitally inputted into a computer-aided design system for scale manipulation before plotting the raster images as feature layers. When a sampling point was selected along a channel, its location was recorded using a global positioning system (GPS) machine (GPSMAP® 60CSx, Garmin International, Inc., Olathe, Kansas, USA) to represent the macrohabitats in the surrounding environment. The coordinates of the habitats containing mosquito larvae were later incorporated into a geographic information system (GIS). We estimated the distance from the breeding sites to human habitation using a GIS tool in order to evaluate the relevance of mosquito abundance and distribution in the study area.

### Habitats along the river channels and tributaries

All the sampling points along the river and stream tributaries were investigated and habitats along the channels were described accordingly. When a sampling point was selected along a channel, its location was recorded using a GPS machine to capture the macrohabitats in the surrounding environment.

### Larval sampling in rivers and streams

Larval sampling of anopheline, culicine, and any other mosquito species was done for all potential breeding habitats within a distance of 50 m from the river. However, sampling in the river or stream was restricted to 10-m radius and 100-m long intervals along the banks, respectively. Each sampling site was dipped 20 times using a standard mosquito dipper of 350 ml (Model 1132BQ, BioQuip Company, California, USA) and abundance was estimated as the total number of mosquitoes recorded in 20 dips per site. However, for breeding sites that were rock pools with narrow openings, a pipette was used to sample as many mosquito larvae as possible. These sites were classified based on the habitat characteristics and the vegetation present along their banks, which mainly comprised of tall and short vegetation. These were described separately, as previous research established that vegetation type influenced the occurrence of different mosquito larvae more so than the malaria-transmitting vectors [16].

Various other habitats adjacent to the river and streams were also sampled. These included open habitats created by the residents to collect water for domestic and agricultural purposes, rock pools, and riverbeds. The vegetated habitats included those supplied by river seepage and swamps adjacent to it.

### Terrestrial habitats away from the river or stream environment

To compare the diversity and abundance of mosquitoes, terrestrial habitats were also sampled. These habitats were situated approximately 15 m away from the main water body. The habitats, mainly containing stagnant water, were grouped as follows: drainages, such as burrows made to supply water to agricultural farms, and hoof prints that were mainly made by wildlife that live in the Maasai Mara National Park.

When water bodies were observed in the distinct macrohabitats, they were grouped based on several characteristics: size, type of vegetation cover, and type of aquatic ecosystem (e.g. swamps, open sunlit puddles, vegetated pools, rock pools, drainages, animal hoof prints, and dams). They were further classified into three categories: habitats with tall vegetation, habitats with short vegetation, and open habitats.

The sampled immature stages of mosquitoes in these habitat types were immediately preserved in 90% ethanol in the field. Upon reaching the laboratory, the specimens were immediately transferred to 99% ethanol. Two hours after the first fixation, specimens were again transferred to fresh 99% ethanol and stored for further identification.

### Species identification

All mosquito larvae were identified to genus and/or species level. Pupae were recorded but were not identified due to a lack of standard identification keys. Larvae belonging to the *An. gambiae s.l*. and *An. funestus* group were further identified by polymerase chain reaction (PCR) and electrophoresis techniques, as described by Cornel and Collins [24]. The primers specific to *An. gambiae s.s*, *An. funestus s.s*, *An. rivulorum*, and *An. arabiensis* mosquitoes were available for the PCR experiment.

### Statistical analyses

All variables were first explored for their distribution and the homogeneity of variance checked using histograms and dot charts. The mean mosquito larvae per habitat type were compared using analysis of variance and chi-square tests (*χ*2). A generalized linear mixed model (GLMM), with date and site code as random effects, was used to identify the most important variables that impact on larval density. The independent variables were: water physico-chemical parameters (pH, conductivity, turbidity, alkalinity, and water hardness) and distance to the nearest human habitation in meters. The forward-backward stepwise model selection method using Akaike’s information criterion was used to select the most appropriate (significant) model [25]. Multicollinearity was assessed by means of a variance inflation factor (VIF). The VIF was calculated for all independent variables, with those showing the greatest values removed from the model. As a rule of thumb, if the VIF value ranges between 5 and 10, multicollinearity is considered present and the variable dropped from the model [26]. The homogeneity of variance was examined using histograms, dot chats and by plotting residuals of every model against its respective predictors. To check the existence of a trend in the spatial distribution of mosquito larvae, we applied the Cox-Stuart test with significant level of α = 5% and taking 200 observations, getting c100 and *n* = 100. The pairs with positive signs were signified as Zi < Zi + c, where T_2_ = 99, and the negative pairs were Zi < Zi + c, where T_1_ = 1.

### Biodiversity indices

The Shannon -Wiener diversity index (H^1^) provides important information on the rarity and commonness of species in a community [27], while Shannon evenness index is a dimension of diversity that defines the number of individuals from each species in the same area [28]. The Shannon index accounts for both abundance and evenness of the species present [29]. It was also used to compare the degree of biodiversity of mosquitoes between the river edge and adjacent terrestrial habitats. Differences in diversity indices were compared using the *t*-test. The Shannon-Wiener diversity index (H^1^) and Shannon evenness index (E) were determined as follows:

Shannon-Wiener diversity index: **H**^**l**^ **= −Σ [(ni / N) x (ln ni / N)]**,

where: **H**^**l**^: Shannon diversity index.

**ni**: number of individuals belonging to i species.

**N**: total number of individuals.

Shannon evenness index: **E = H/log(S)**,

where: **E**: evenness index.

**H**: Shannon diversity index.

**S**: species number.

## Results

### Mosquito populations

The number of mosquito-specific larvae species collected during this survey is shown on Table 1. A total of 4001 mosquito larvae were captured and identified. Of the 4001 mosquitoes collected, *An. gambiae s.s*. comprised 1038 (25.9%) of the total, while 973 (24.3%) were *An. arabiensis* mosquitoes identified using the PCR technique. The top three most dominant mosquito species were *An. gambiae s.l*. (50.2%), *Culex* spp. (29.5%), and *An. coustani* complex (8.0%). Other species found included: *An. maculipalpis* (3.6%), *An. funestus s.s,* (3.5%), unidentified *An. funestus* (1.3%), *An. azamiae* (1.1%), *An. pharoensis* (1.1%), *An. hamoni* (0.7%), *An. christyi* (0.3%), *An. ardensis* (0.05%), *An. faini* (0.02%), *An. sergentii* (0.02%), *An. rivulorum* (0.4) and *Aedes* spp. (1.3%).

**Table 1:** Mosquito species, their numbers and percentage composition within the Mara river basin

Mosquito species	No. mosquitoes	% composition
*An. gambiae s.s.*	1038	25.9
*An. arabiensis*	973	24.3
*Cx. quinquefasciatus*	761	19.0
*Cx. pipiens* complex	420	10.5
*An. coustani* complex	321	8.0
*An. maculpalpis*	145	3.6
*An. funestus s.s.*	140	3.5
Unidentified *An. funestus*	50	1.3
*An. azamiae*	45	1.1
*An. phaorensis*	44	1.1
*An. hamoni*	28	0.7
*An. rivulorum*	15	0.4
*An. christyi*	12	0.3
*Aedes* spp.	5	0.1
*An. ardensis*	2	0.05
*An. faini*	1	0.02
*An. sergeti*	1	0.02
Total	4001	100.0

### Mosquito species abundance per habitat type

The habitats surveyed included those that were in the selected points along the main Mara River, which included drying streams of the Mara River tributaries and their feeder streams, swamps along the banks and adjacent habitats, open sunlit puddles, rock pools, springs, dams, livestock hoof prints, vegetated pools by the river/streams, and those in the terrestrial habitats and drainages.

The mean *An. arabiensis* larvae were highest in the drying streams (μ = 34.6, SE = 23.8), followed by open puddles (μ = 29.1, SE = 12.1), drainages with scattered grass (μ =12.3, SE = 6.4), and vegetated pools (μ = 8.8, SE = 1.4), as compared to *An. gambiae s.s.* larvae in those habitats (μ = 27.4, SE = 18.4; μ = 26.2, SE = 4.1; μ =19.1, SE = 3.6, respectively; *P* ≤ 0.01). Swampy habitats on the edges of streams and rivers covered by grass were mainly dominated by mosquitoes of the *An. funestus* group. However, the above three species were also found in pools covered by debris of different types (other habitat types). The presence of *An. caustani* mosquitoes was found to be significantly higher in swampy habitats (μ = 48.17, SE = 8.73, *P* ≤ 0.01) than in any other habitat type. *An. ardensis*, *An. faini*, *An. hamoni*, and *An. sergentii* populations were the lowest compared to all other populations sampled. *An. maculipalpis* larvae were found mainly in open sunlit habitats (brick making puddles which are formed on land from the actual process of making bricks from soil), with significantly higher populations than in any other sampled habitats (see Table 2).

**Table 2:** Mean densities ± SE different larvae of mosquitoes (per habitat type) along the Mara River

**Taxa**	Drying streams	*Other habitat types	Open puddles	Vegetated pools	Rock pools	Animal hoofprints	Rivers
*An. arabiensis*	34.6 ± 23.8	12.31 ± 6.4	29.1 ± 12.1	8.8 ± 1.3	4.3 ± 0.2	4.1 ± 5.1	0.0
*An. gambiae s.s.*	27.4 ± 18.4	20.3 ± 5.0	26.2 ± 4.1	19.1 ± 3.6	0.0	2.1 ± 1.3	0.0
*Cx. pipiens*	12.2 ± 7.4	3.8 ± 0.8	16.2 ± 1.4	5.1 ± 0.9	0.0	4.6 ± 1.5	0.0
*An. coustani*	20.3 ± 9.7	48.2 ± 8.7	1.0 ± 0.4	11.0 ± 4.4	0.0	0.0	0.0
*Cx. quinquefasciatus*	20.2 ± 7.6	27.2 ± 0.2	18.1 ± 8.0	22.1 ± 0.1	0.3 ± 0.2	0.1 ± 0.1	0.0
*An. funestus* group	0.9 ± 1.2	0.0	0.0	5.6 ± 1.5	0.0	0. 0	0.0
*An. pharoensis*	2.1 ± 0.1	5.2 ± 1.2	0.0	0.0	0.0	0.0	0.0
*An. azamiae*	0.0	0.1 ± 0.1	0.2 ± 0.1	<0.1	0.0	0.0	0.0
*An. christyi*	0.3 ± 0.1	0.3 ± 0.1	0.0	<0.1	0.0	0.0	0.0
*An. maculipalpis*	0.0	<1.0	26.3 ± 11.4	0.1 ± 0.1	0.0	0.0	0.0
*An. ardenis*	0.0	0.0	2.2 ± 1.3	0.0	0.0	0.0	0.0
*An. sergentii*	1.4 ± 0.4	2.3 ± 0.1	0.1 ± 0.1	0.0	0.0	0.0	0.0
*An. faini*	0.0	0.0	3.4 ± 1.4	1.6 ± 0.5	0.0	0.0	0.0
*Aedes* spp*.*	0.0	0.0	< 0.1	0.0	0.0	0.0	0.0

The numbers of mosquitoes were higher in the drying streams followed by isolated swamp by the river. Except for only two *An. coustani* caught in the river environment, none of the mosquito larvae species was sampled from the main Mara River

Were higher abundances of most mosquito species, including *An. gambiae s.l*, *An. funestus* group, *An. pharoensis*, *An. ardensis*, *An. azamiae, An. christyi*, *An. maculpalpis*, *An. hamoni*, and *An. sergentii*, in habitats with short grass compared to habitats with tall grass and open sunlit habitats. Only *Culex* spp. and *An. faini* mosquitoes were more abundant in open sunlit pools than in habitats with short grass and vegetation with tall grass.

### Terrestrial versus river edge habitats

Mosquito larvae were found inhabiting both the terrestrial and river edge habitats. The total number of mosquitoes collected at the terrestrial habitats was 1289, while those collected at the river edge habitats was 2712.

River edge habitats had a total of 170 mosquito breeding sites sampled, while terrestrial habitats had 90 such sites sampled. At the river edge, 87 (51.2%) of the pools were drying streams, while 49 (28.8%) were made up of large swamps. The remaining 34 (20.0%) were mainly rock pools and small patches of puddles. The aquatic habitats within the river environment were further classified according to vegetation height: tall plants (> 1 m), short plants (< 1 m, mainly grass and sedge or floating vegetation), and open sunlit pools (see Table 3).

**Table 3:** Percent composition of mosquito larvae species collected at various habitat types based on vegetation characteristics

Mosquito species	Short grass (%)	Tall grass (%)	Open sunlit (%)	Total (%)
*An. gambiae s.l.*	618 (39.1)	2 (0.1)	959 (60.8)	1579 (100)
*An. funestus* group	65 (81.2)	15 (18.8)	0(0.0)	80 (100)
*An. coustani*	299 (53.6)	255 (45.7)	4 (0.7)	558 (100)
*An. pharoensis*	40(62.5)	22 (34.4)	2 (3.13)	64 (100)
*Culex* spp.	620 (41.5)	37(2.5)	837 (56.0)	1494 (100)
*An. ardensis*	2 (66.7)	1(33.3)	0 (0.0)	3 (100)
*An. azamiae*	40 (76.9)	0(0.0)	12 (23.1)	52 (100)
*An. christyi*	8 (66.7)	3(25.0)	1 (8.3)	12 (100)
*An. maculipalpis*	75 (57.5)	0(0.0)	55 (42.3)	130 (100)
*An. hamoni*	15 (53.6)	0(0.0)	13 (46.4)	28 (100)
*An. sergentii*	1(0.0)	0(0.0)	0 (0.0)	1 (100)
*An. faini*	0 (0.0)	0(0.0)	1 (100)	1 (100)
Total	1783	335	1884*	4001

*Habitats with open sunlit produced the highest number of mosquitoes overall

Overall, a total of 180 habitats were surveyed based on their vegetation height. Of the total 1783 mosquitoes sampled in the habitats with short vegetation, 34.7% were *An. gambiae s.l.*, 3.6% were of the *An. funestus* group, 16.8% were *An. coustani*, 2.2% were *An. pharoensis*, 34.8% were *Culex* spp., 0.1% were *An. ardenis*, 2.2% were *An. azamiae*, 0.5% were *An. christyi*, 4.2% were *An. maculipalpis*, 0.8% were *An. hamoni*, and 0.1% were *An. sergentii*. *An. faini* was non-existent in short grass habitats. There were significant differences in mosquito larvae species abundance among different habitat types (*χ*^*2*^ = 893.97, df = 2, *P* ≤ 0.01), with *An. gambiae s.l.* larvae being most prominent in open sunlit pools (60.7%) as compared to short grass habitats (39.2%) and tall grass habitats (0.1%). Similarly, there was a significant difference in the abundance of mosquitoes belonging to the *An. funestus* group among different habitat types (*χ*^*2*^ = 86.875, df = 2, *P* < 0.01), with this group dominating habitats with short grass (81.2%) as compared to habitats with tall grasses (18.8%) and open sunlit pools (0.0%). *An. coustani* larvae were highest in short grass habitats (53.6%) as compared to tall grass habitats (45.7%) and open sunlit pools (0.7%); with a significant difference observed (*χ*^*2*^ = 272.33, df = 2, *P* < 0.01). The trend was similar for the rest of the anopheline species. On the contrary, *Culex* spp. mosquitoes dominated open sunlit pools (56.0%), followed by short grass habitats (41.5%) and then tall grass habitats (2.5%). The differences were statistically significant (*χ*^*2*^ = 687.4, df = 2, *P* ≤ 0.01).

Terrestrial habitats mainly comprised open sunlit pools. A total of 80 microhabitats were sampled and found to be harboring different mosquito species. *Culex* spp. were the most prominent species, accounting for about 58.9% of the mosquitoes collected. The remaining proportion (41.1%) consisted of anopheline species, the majority of which occupied vegetation with littered dry leaves or scattered short grass. The populations of the two main species differed significantly in the terrestrial aquatic habitats (*χ*^*2*^ = 24.012, df = 1, *P* ≤ 0.01).

### Species diversity and evenness

The species diversity and evenness indices were calculated to provide information on mosquito community structure between the river and adjacent habitats. Shannon indices are presented in Table 4.

**Table 4:** Summary of the diversity indices as described by mosquito species richness along the Mara River, Kenya and Tanzania

Indices	Terrestrial	River edge	*P*-value
	Habitats	Habitats	
Species number	1289	2712	–
Species Richness	9	12	–
Shannon-Weinner Diversity Index	1.1747	1.4380	0.342
Shannon Evenness Index	1.2640	2.1332	0.002

Albeit not significant, the Shannon-Wiener diversity index was higher for river edge habitats (1.438) compared to terrestrial habitats (1.1747). However, the diversity index did not vary between aquatic terrestrial habitats and river edge habitats (*t* = 0.3120, df = 1, *P* = 0.342).

**Fig. 2:**
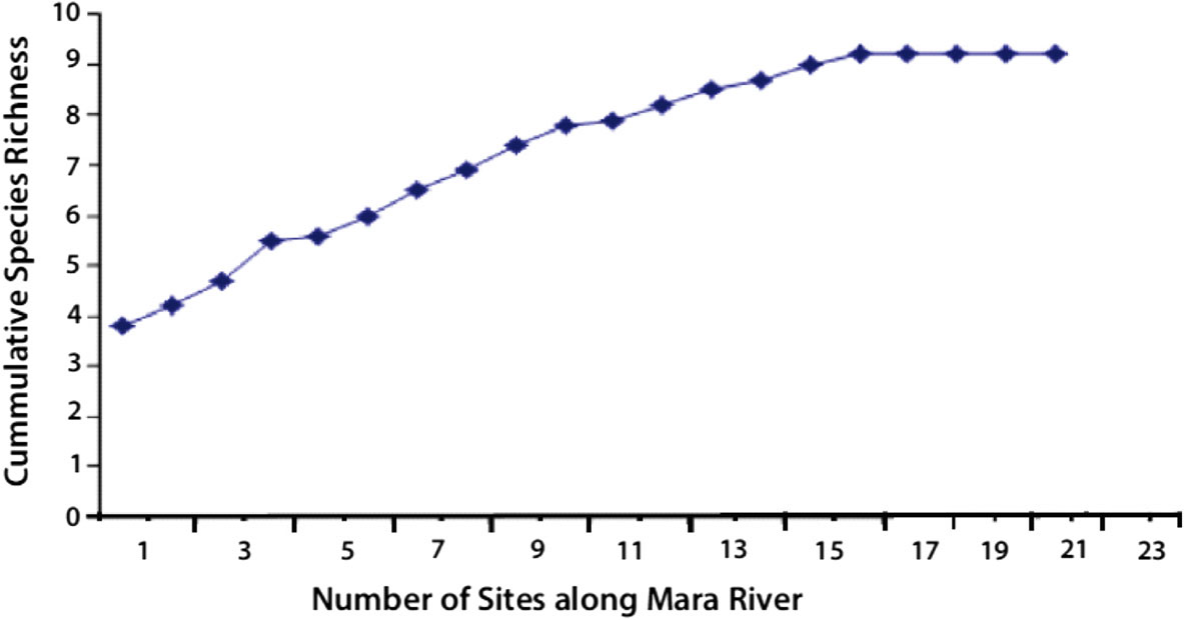
Species richness estimation in all sites along the Mara River

The Shannon evenness index was higher in river edge habitats (2.13) than in terrestrial aquatic habitats (1.26), and a significant difference was observed between the two broad habitat types (*t* = 7.123, df = 1, *P* = 0.002). As the number of mosquitoes increased, the diversity of larval mosquitoes became linear, further demonstrating the diversity of larval mosquitoes and species richness along the Mara River (see Fig. 2).

### Influence of distance and elevation on anopheline and culicine larvae distribution

Most of the breeding habitats were recorded within a distance of 70–450 m from the nearest human habitation, with an average distance of 151.0 ± 8.43 m. However, these measurements were not taken within the national park as human habitation is not permitted there.

**Fig. 3:**
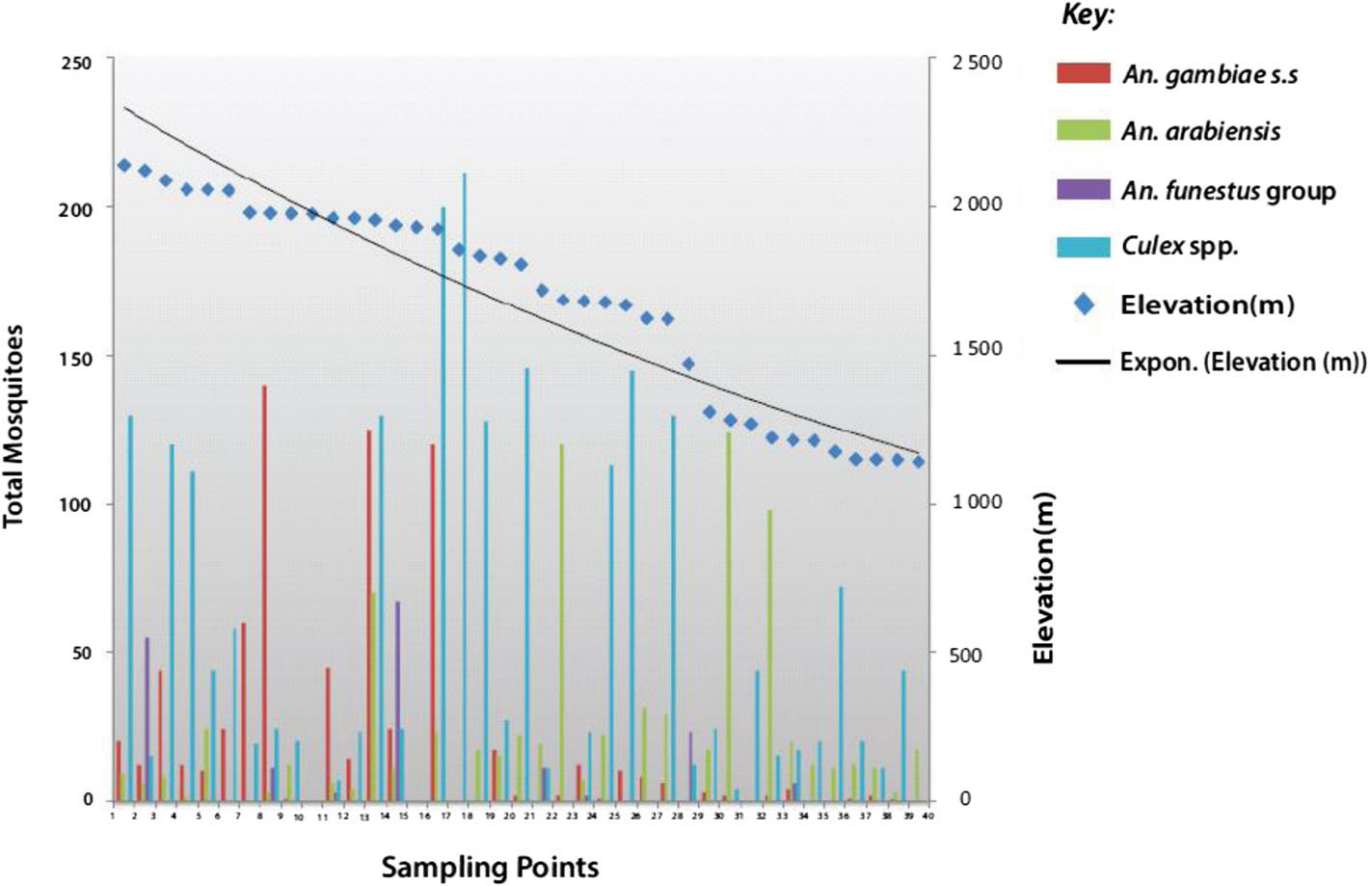
Distribtion of the four main disease-transmitting vectors along the Mara River and its tributaries

**Fig. 4:**
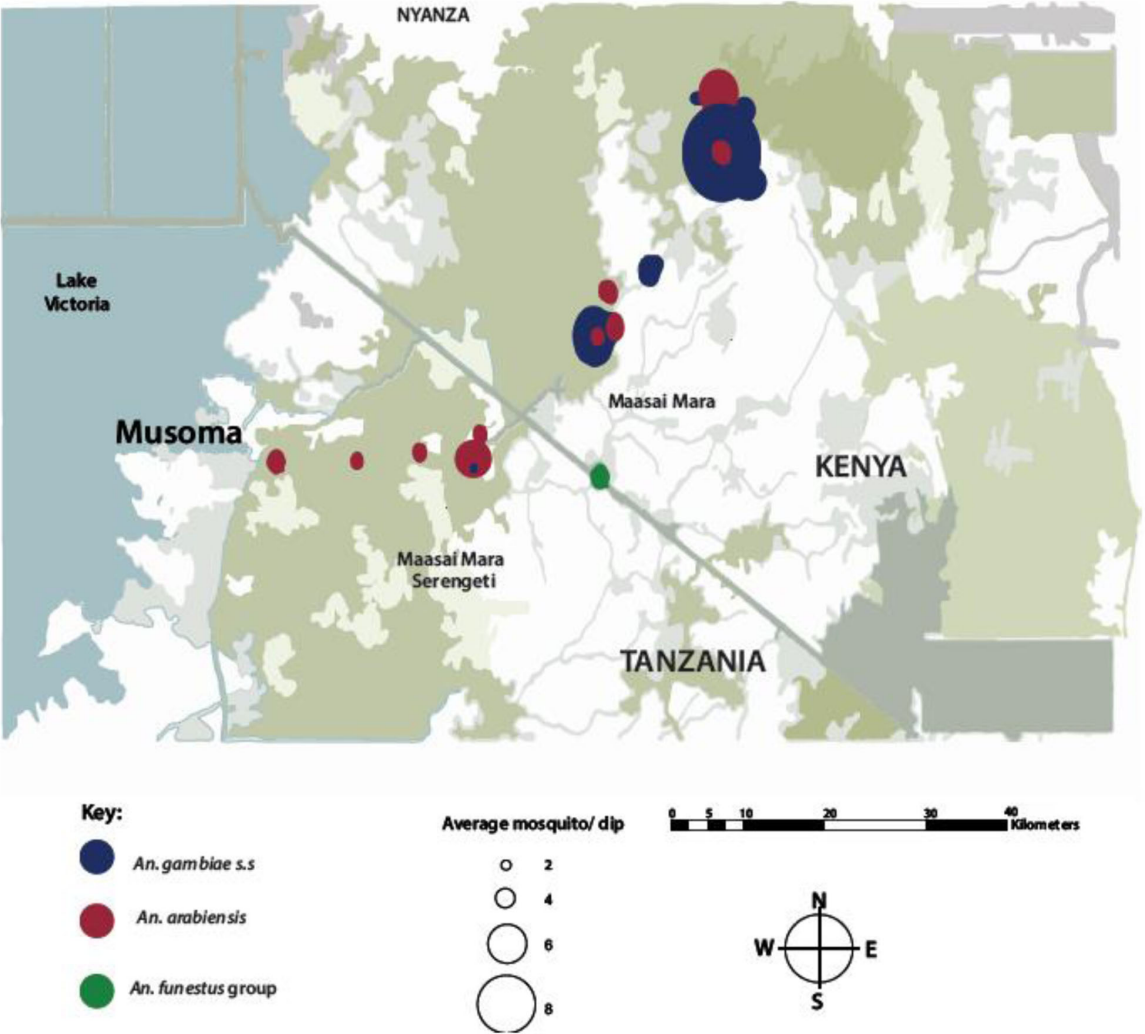
Map of the distribution of the three main malaria vectors along the Mara River. The *red* dots show the spatial distribution of *An. arabiensis* mosquitoes, the *blue* dots show the spatial distribution of *An. gambiae* s.s. mosquitoes, and the *green* dots show the spatial distribution of mosquitoes belonging to the *An. funestus* group

Sampling was conducted at the upper catchment area of the Mara River in Kenya, an area with an elevation of about 2126.2 m above sea level, while the lowest elevation (1147.4 m) was recorded in Kwebuse village in Tanzania. Trend for the four top most important mosquitoes in the area (*An. arabiensis*, *An. gambiae s.s*, *An. funestus* group, and *Culex* spp.) showed that sites with an elevation of below 1700 m, especially those that were located near Tanzania, were favorable for *An. arabiensis* abundance, while those above 1900 m (mainly in the upper catchment of the Mara River) were dominated by *An. gambiae s.s.* mosquitoes. Mosquitoes of the *Culex* spp. were distributed evenly across the study sites. There were small numbers of mosquitoes belonging to the *An. funestus* group and other anopheline species, thus their distribution was not clearly depicted (see Fig. 3). The Cox-Stuart test for trend analysis showed a trend on mosquito larvae distribution (*t* = 17. 283, *P* = 0.001), thus we accepted the hypothesis of a significant trend. Spatial distribution of the collected larvae is presented in Fig. 4.

In August, during the sampling period, 70.9% of the habitats that harbored mosquito larvae were located above an elevation of 1580 m, at the highland site, while 82.4% of breeding habitats that had mosquito larvae were located above an elevation of 1220 m at the lowland site. *An. gambie s.s.* mosquitoes dominated the upper part of the Mara River of Kenya, while *An. arabiensis* mosquitoes showed dominance on the upper side of Tanzania, which is characterized by lower elevation. *Culex* complex had a marked distribution all over the study area. Mosquitoes of the *An. funestus* group, although few, were evenly distributed across the study sites. 

### Variation in the mean physico-chemical water quality parameters in different habitats along the Mara River

Table 5 shows the physico-chemical parameters for each of the eight different habitats. The findings showed that dissolved oxygen (DO) was highest (6.4 ± 0.7 mg/L) in rivers and lowest (2.4 ± 2.7 mg/L) in swamps. Most habitats, however, had DO values ranging between 4.0 and 5.6 mg/L. Conductivity levels across different habitats showed wide variations, ranging between a mean of 144.5 ± 97.6 μS/cm for rivers and 368.0 ± 125.9 μS/cm for rock pools. Dams and streams also recorded relatively high mean conductivity levels of 290.0 ± 186.5 μS/cm and 269.8 ± 213.8 μS/cm, respectively. The pH levels varied only slightly between different habitats, ranging between 7.0 and 8.2. Only swamps recorded a mean pH of 7.0 ± 1.3 (neutral), while other habitats recorded alkaline pH, i.e. slightly above 7.0. Turbidity levels, varied highly between different habitats within the Mara River basin, with the highest mean turbidity of 542.6 ± 2.3 Nephelometric turbidity unit (NTU) recorded in rock pools and the lowest mean turbidity of 95.2 ± 131.9 NTU recorded in dams. Mean alkalinity and hardness were both highest (400.0 ± 282.8 mg/L and 372 ± 393.2 mg/L, respectively) in drainages. However, the lowest mean alkalinity (100.0 ± 62.4 mg/L) was recorded in dams, while the lowest mean hardness (58.5 ± 46.7 mg/L) was recorded in swamps. There were slight variations in temperature between different habitats within the Mara River basin, ranging between 19.7 ± 2.3 °C in the main river to 26.2 ± 3.4 °C in rock pools. Only swamps recorded slight salinity of 0.4 mg/L, while all the other sites had zero (0) salinity.

**Table 5:** Average physicochemical parameters at different mosquito larvae habitats along Mara River basin

Habitats (No. of sites)	DO (mg/L)	pH	Alkalinity (mg/L)	Hardness (mg/L)	Turbidity (NTU)	Conductivity (μS/cm)	Temperature (°C)	Salinity (mg/L)
Dam	4.7 ± 1.8	8.1 ± 0.4	100 ± 62.4	87.7 ± 56.2	96.9 ± 142.0	269.8 ± 213.8	24.4 ± 1.9	0.0 ± 0.0
Stream	5.3 ± 1.6	8.1 ± 0.6	126.2 ± 26.5	102.4 ± 68.9	124.3 ± 152.6	290 ± 186.5	22.5 ± 2.1	0.0 ± 0.0
Swamp	2.4 ± 2.7	7.0 ± 1.3	244.5 ± 274.6	58.5 ± 46.7	142.2 ± 108.5	174.3 ± 59.2	23.2 ± 4.9	<0.1
Drainage	4.3 ± 3.8	7.3 ± 0.5	400 ± 282.8	372 ± 393.2	144.8 ± 84.3	168.5 ± 13.4	24.2 ± 0.7	0.0 ± 0.0
Rock pool	6.0 ± 0.7	7.1 ± 0.8	153 ± 60.8	127 ± 69.3	542.6 ± 2.3*	368.0 ± 125.9*	26.2 ± 3.4	0.0 ± 0.0
Puddles	5.6 ± 0.8	8.2 ± 0.5	104 ± 103.0	188 ± 247.7	95.2 ± 131.9	168.8 ± 87.3	25.2 ± 2.3	0.0 ± 0.0
Spring	4.0 ± 0.3	8.3 ± 0.6	124 ± 113.2	183 ± 148.4	134.5 ± 121.7	155.7 ± 88.4	26.3 ± 2.2	0.0 ± 0.0
River	6.4 ± 0.7	7.3 ± 0.4	100 ± 199.2	178 ± 228.8	135.2 ± 142.4	144.5 ± 97.6	19.7 ± 2.3	0.0 ± 0.0

*There were highly elevated levels of turbidity and conductivity in rock pools, probably due to accumulation of dissolved particles

### Relating physico-chemical parameters and distance to mosquito larvae abundance using the GLMM

A GLMM analysis conducted relating physico-chemical parameters to mosquito larval density found that conductivity, DO, temperature, and turbidity were the most favorable water factors for immature mosquito survival (see Table 6). The results further indicated that distance to the nearby human habitation was another important factor influencing mosquito larvae abundance.

**Table 6:** GLMM relating mosquito larval density to physico-chemical parameters and distance along the Mara River

Variable	Estimate	SE	Z	*P*-value
(Intercept)	3.27	0.65	5.05	<0.001
Conductivity	0.02	0.05	3.68	<0.000
Distance	0.01	0.03	4.57	<0.001
DO	0.31	0.04	7.37	<0.001
Temperature	0.08	0.02	7.65	<0.001
Turbidity	−0.03	0.07	−5.25	<0.001

## Discussion

Most mosquito larvae were found in isolated pools created by receding waters or in temporary habitats near the Mara River, or along the perennial Mara River tributaries of Amala and Nyangores. Most of these habitats were characterized by various types of vegetation, which provided ideal microhabitats for mosquitoes, especially *An. coustani* and those belonging to the *An. funestus* group*.* These species were found in higher densities in swampy vegetated areas. However, several patches of open sunlit pools adjacent to the main Mara River were dominated mainly by *An. gambiae s.l*. and *An. maculipalpis* mosquitoes. In terms of malaria transmitting vectors, *An. gambiae s.l.* was the most dominant species in the samples collected from the open sunlit pools.

A study conducted by Minakawa et al. [30] suggested that *An. gambiae s.l.* mosquitoes tend to prefer open sunlit pools, as was also evident in the current study. The same study further reported that development of *An. gambiae* mosquito larvae ceases at temperatures below 16 °C and that they die at temperatures below 14 °C. Paaijmans et al. [31] and Munga et al. [32] also reported that temperature affects the rate of larval development, while Tuno et al. [33] reported that high temperatures influence pupation rates as well as larval survivorship. Afrane et al. [34] also reported that larval-to-adult survivorship and larval-to-adult development times are influenced by temperature.

Of the 4001 mosquitoes collected, *An. gambiae s.s*. comprised 1038 (25.9%) of the total, while 973 (24.3%) *An. arabiensis* mosquitoes were found using the PCR technique. Other *Anopheles* spp. mosquitoes that did not belong to *An. gambiae s.l*. also existed in the study sites. However, these species failed to amplify with primers designed for *An. gambiae* mosquitoes. Therefore, future studies should consider identifying all species that belong to the same genera in the study area using oligo primers specific to all the different sibling species.

In the Mara River basin, mosquitoes of the *An. funestus* group were mostly found in swamps and puddles covered with short grass, while *An. pharoensis*, *An. azamiae*, *An. christyi*, *An. maculipalpis*, *An. hamoni*, and *An. sergentii* mosquitoes dominated open sunlit puddles, hippo hoof prints, and drainages. Among these species, *An. pharoenis* and *An. azamiae* have been reported as malaria vectors in Ethiopia and Cameroon [35]. In other areas, *An. gambiae s.l.* mosquitoes have also been found in high abundance, either in temporary sunlit pools or open habitats with scattered short grass [30, 36]. Similarly, mosquitoes of the *An. funestus* group mainly prefer swamps along the shores of Lake Victoria, Western Kenya [16], and are hardly found inhabiting open sunlit pools. This variability in species abundance at the two sites may be attributed to local ecological differences.

At the Transmara border site, the habitats were mainly rock pools with stagnant water created by the hydrologic effect of stream water that hits the riverbanks and settles on pocket-like rocky habitats. The water in these habitats was clearer and was shielded from direct sunlight. Consistent with the current findings, Munga et al. [32] also reported that the presence of *An. gambiae s.l.* and *An. funestus* complex mosquitoes in natural aquatic habitats in the Western Kenyan highlands was inversely related to canopy cover. Similarly, Minakawa et al. [6] and Fillinger et al. [37] reported that sunlit pools are most preferred by *An. gambiae s.l.* mosquitoes. It is recommended that these habitats be closely monitored if the risk of malaria transmissions is to be reduced among the riparian communities.

The Mara River is perennial, flowing all year round, with levels of water fluctuating during the dry and rainy seasons [36]. As a result, small pools of water are created by the riverside during the rainy season, which dry progressively as the rainfall recedes [36]. During this time, these drying pools create suitable breeding habitats for *Anopheles* mosquito. The observation that *An. gambiae s.l*. populations were abundant in drying stream tributaries is a clear confirmation that malaria vector species prefer breeding in stable waters with minimal disturbance both in terrestrial located habitats and river/stream habitats, as was also reported by Ageep et al. [38].

In terrestrial habitats, open sunlit puddles were found to be harboring more mosquitoes as compared to roadside ponds with vegetation. In the river habitats, more mosquitoes were found in slow flowing streams and riverbeds with little vegetation as compared to open water, an indication that aquatic vegetation plays an important role in harboring malaria transmitting vectors.

This study also shows that *Culex* spp. were the most widespread mosquito larvae along the Mara River basin, as they were collected from a variety of habitats. This is a clear indication that *Culex* spp. larvae have a greater degree of adaptability to different habitats than other mosquitoes. The presence and wide distribution of *Anopheles* spp., the vector of human malaria, constitute a major potential health problem. *Anopheles* spp. particularly differ in host-seeking behavior, with some species preferring to feed on humans, while others feed on animals. Mosquito feeding behavior is therefore considered a primary requisite for understanding the transmission of malaria [39]. Further studies on the vectorial capacity of these disease pathogen vectors are required and every effort should be made to prevent their spread within the Mara River basin.

In this study, most mosquito larvae were collected from water accumulations with different degrees of turbidity. Post and Kwon [40] attributed the favorable effect of sunlight on mosquito larval population. These scholars [40] further noted that production of algae which form favourable larval food and are important in maintaining the balance of dissolved gases also requires sufficient amount of sunlight. Kenaway and El-Said [41], however, reported that turbidity had no significant effect on *Culex* spp. larvae; though habitats that were shaded, vegetated, and had stagnant water were generally preferred for larval breeding.

Both anopheline and culicine larvae were positively associated with DO. Previous reports also indicate similar association between *Cx. quinquefasciatus* and *An. arabiensis* larvae with DO [32]. Oyewole et al. [42] concurred that optimum DO might have contributed to the survival and breeding of *Anopheles* larvae in the Mara River. It has also been observed that DO saturation decreases when the bed sediment changes from stony substratum to soft sediments [43, 44]. Human settlements, urbanization, and other pressures can influence changes in the water chemistry, as well as reduce DO levels [45]. These results are in line with the findings of Kasangaki et al. [46], who reported that clearance of forests was endangering freshwater ecosystems in East Africa.

*Culex* spp*.* and *Anopheles* spp. larvae showed positive association with conductivity. However, unlike in the current study, Dejenie et al. [47] reported a negative association between conductivity and *Cx. quinquefasciatus* larvae presence in Tigray micro dams in Ethiopia.

Removal of riparian vegetation has also been reported to modify stream hydraulics, substrate features, light and thermal system, water chemistry composition, and organic matter contribution, all of which affect riverine communities [48, 49]. Based on the findings of this study, the two most important factors found to influence the abundance and distribution of different mosquito species within the Mara River basin were habitat type and water chemistry. Ecological disturbance resulting from altered land use in the highland regions was initially reported as a possible cause for the puzzling increase in highland malaria [50]. Although larval abundance is only one factor influencing the subsequent vector-biting rate and malaria transmission, reductions in malaria cases have been observed after large-scale implementation of larval control measures [37].

Chemical composition of water influences larval species and their populations. For instance, *An. merus* and *An. melas* mosquitoes, both members of *An. gambiae* complex, were shown to breed in salt water with a pH greater than 7.0 [35]. A similar finding was also reported by Dejenie et al. [47] in Ethiopia. However, in Mbita Point, Western Kenya, water pH did not determine the breeding of anopheline mosquitoes [48]. In the current study, although the effect of pH was not evident, a relationship between DO, temperature, conductivity, turbidity, and mosquito larvae was observed.

Turbidity of water has been reported to have an effect on larval populations by influencing adult oviposition behavior. Laboratory studies have further demonstrated that chemoattractants from decaying organic matter may also play a role in the oviposition behavior of gravid *An. gambiae* mosquitoes [49]. In the current study, mosquito abundance showed a negative significant association with water turbidity, suggesting that mosquitoes prefer clearer water. Thus, our results contradict those of McCrae [50] and Kenaway and El-Said [41], which showed that some mosquitoes prefer turbid to clearer water for oviposition. However, other studies reported that *Culex* spp. mosquitoes survive more in turbid water than *Anopheles* spp. mosquitoes [51, 52].

In the current study, we encountered many small streams and water bodies, and larval surveys of these bodies revealed the presence of anopheline larvae at all altitudes. However, we observed variation in the occurrence of anopheline sibling species, for instance, higher number of *An. gambiae s.s*. mosquitoes were recorded in the higher altitudes of Rift Valley in Kenya, but their numbers declined upon approaching the lower altitude regions in the western side of Tanzania. Just as in the upper catchment areas, the western parts of the Mara have lost considerably more acres of forest, leaving most parts of the stream bare. *An. gambiae s.l*. mosquitoes tended to inhabit temporary freshwater pools in cleared areas resulting from deforestation such as the forest fringe, as observed in the present study.

We estimated the distance from all breeding sites to human habitation in order to deduce the public health importance of this study. Previous studies have associated distance from breeding sites to human habitation with an abundance of mosquitoes, particularly malaria vectors [30, 53]. This is explained by the fact that most malaria vectors alternate between their vertebrate hosts and stagnant water bodies for blood meal and oviposition sites, respectively. These two resources are obligatory requirements for completion of the mosquito gonotrophic cycle [54]. In the current study, the distances between breeding sites and human habitation ranged from approximately 50 m and 450 m. According to Greenberg et al. [55], most mosquitoes stay within a mile or two of their source. However, some have been recorded as far as 75 miles from their breeding source [56]. Carter et al. [57] observed that malaria vectors have a typical flight range of 1–2 km, which is an essential component that influences their distribution. They further reported that a limited energy reserve restricts *An. gambiae* mosquitoes from long-range flights to lay eggs in aquatic habitats far from human habitations.

The current study shows that more than 70% of the breeding sites were located within 200 m of human habitation. Minakawa et al*.* [30] reported that 90% of *An. gambiae* mosquitoes were found to breed within 300 m of human habitation, while Shililu et al. [58] and Carter et al. [57] reported that *An. gambiae* mosquitoes prefer small sunlit pools and man-made habitats in the vicinity of human habitations. This suggests that human host availability may affect the relative abundance and distribution of mosquito larvae in aquatic habitats. According to Killeen et al. [59], availability of preferred hosts for blood meal within the flight range of malaria vectors influences the emergence rate, feeding cycle length, malaria transmission dynamics, and even the survival of the vectors. According to Chaves et al. [60], *An. gambiae s.s.* mosquitoes prefer feeding mostly on humans even when introduced to other hosts under field trials, while Mahande et al. [61] reported that *An. arabiensis* mosquitoes prefer feeding on animals. Hoek et al. [62] suggested the use of a distance of 750 m as a cut-off point for developing a risk map of malaria in Sri Lanka.

The suitability of available aquatic habitats within the vicinity of human habitations therefore determines adult vector populations and the risk of malaria transmission in a locality [63]. Findings from this study suggest that both biotic (flora and fauna) and abiotic (chemical and physical) factors play a significant role in larval habitat preference by both *Culex* spp*.* and *Anopheles* spp. mosquitoes. Thus, such factors should be taken into consideration when designing an integrated vector control program. Further, cross-sectional studies of aquatic mosquito larvae breeding habitats and non-breeding habitats are recommended; including taking into account all biotic and abiotic variables using accurate quantitative measurements. Abundance of *Culex* spp*.* and *Anopheles* spp. mosquitoes showed a positive association with conductivity. However, unlike our findings, Burke et al. [64] reported a negative association between conductivity and *Cx. quinquefasciatus* larval presence in Puerto Rico. Shililu et al. [58] concluded that the distribution of mosquitoes in different habitats is a result of more complex interactions of different habitat factors.

The main river had no mosquitoes. However, large swamps with tall emergent vegetation adjacent to the Mara River were found to harbor only *An. coustani* mosquitoes. The many habitats adjacent to the main river created through human activities, such as brick making or animal activities especially at watering points, appeared to harbor most malaria transmitting vectors, i.e. *An. gambiae s.s.* The receding river and stream water levels caused by the destruction of forests and rock pools that initially were below the water surface, especially during dry spells, seem to be good breeding microhabitats for *An. gambiae s.l*. mosquitoes, while puddles covered with vegetation are suitable for mosquitoes of the *An. funestus* group*.* Therefore, these conditions are potentially improving the habitat diversity for mosquito vectors, which are good indicators of the health of riverine ecosystem. Barros et al. [65] observed that mosquito reproduction is successful only if larval habitats remain stable for a duration equivalent to the development of immature stages.

In the current study, open sunlit puddles, rock pools, and drains, which produced high numbers of mosquitoes, were shallow, isolated, and tended to limit predator access. Such habitats were then perfect breeding sites for potentially harmful mosquito species, some of which are known carriers of malaria parasites.

This study compared terrestrial water pools with those adjacent to the river, as past studies have suggested that pools along shores are more productive than terrestrial water pools [6]. Quantification of diversity in this way was meant to help understand the mosquito community structure. In the current study, albeit not significant, the Shannon diversity index was slightly higher for river edge habitats (1.4380) compared to terrestrial habitats (1.1747), although both were still low considering that the typical value of the index ranges from 1.5 (low species richness and evenness) to 3.5 (high species evenness and richness) [28]; however, values beyond these limits up to a maximum of 5 may also be encountered. The evenness index was higher in river edge habitats (2.13) than terrestrial aquatic habitats (1.26), reflecting a variation in abundance of mosquito species between the two sites along the Mara River. A generally low species diversity was observed in the current study irrespective of the habitat type, implying that the habitats were not particularly suitable for a large number of different species. However, the evenness index showed significant differences between river edge habitats and terrestrial habitats, with the river edge habitats having slightly higher evenness values than terrestrial habitats. This implies that the river edge habitats are probably more stable given the longer duration of time that water is likely to stagnate there making them suitable for mosquito breeding compared to terrestrial habitats.

Considering that similar proportions of all sub-species give an evenness index of 1, with higher values reflecting very dissimilar proportions (some rare and some common species), it is apparent that mosquito sub-species were clearly dismal as reflected by the dominance of *An. gambiae s.l*. and *Culex* spp. mosquitoes over other mosquito species in both habitats. This could be an indication that some species are better adapted than others to the habitats sampled.

### Limitations of the study

This study was designed and conducted during the dry period along the Mara River and its tributaries in Kenya and Tanzania. From our study, multiple variables were measured including those driven by different climatic factors. We therefore acknowledge the limitations of a cross-sectional study in which all aspects of habitat parameters, especially changes in the physicochemical parameters over time (including seasonality) are not taken into consideration. In addition, the vectorial capacity of the various mosquito species collected should have been carried out so as to be able to make more concrete conclusions on malaria transmission within the Mara River basin.

## Conclusions

This study found that rock pools and drying or slow moving streams with low water levels, sections of exposed riverbeds, and swampy areas with vegetation were particularly ripe for the development of mosquito larvae compared to other habitat types. These habitats are critical during dry periods when the stagnant water becomes most suitable for mosquito breeding, and may play an important role in local malaria transmission during such periods.

The results of this study also showed that most *Anopheles* spp. mosquitoes are widely distributed in the Mara River basin and, more interestingly, that they could be relying on nearby human settlements and perhaps presence of livestock and wildlife in close proximity to their breeding sites for their blood meal.

We recommend that further longitudinal studies are conducted on changing physical and chemical water characteristics taking seasons, and human and livestock presence into account, and how these influence mosquito abundance in different habitat types. This will shed more light on the Seasonal dynamics of mosquito abundance and malaria transmission within the Mara River basin. Given the length of the Mara River coupled with its many tributaries, suitable aquatic microhabitats that include rock pools, swamps, and open sunlit puddles are likely to be extensive, subsequently supporting huge numbers of mosquitoes, which are then likely to enhance the transmission of malaria not only within the Mara River basin but also in parts of the larger Lake Victoria basin. Therefore, vector control programs targeting potential habitats identified as the most likely breeding sites for mosquitoes will be of great benefit for Mara River basin inhabitants.

## Additional file

Additional file 1: Multilingual abstracts in the six official working languages of the United Nations. (PDF 673 kb)

## Abbreviations

DO: Dissolved oxygen
GIS: Geographic information system
GLMM: Generalized linear mixed model
GPS: Global positioning system
PCR: Polymerase chain reaction
VIF: Variance inflation factor
