# Supplementary material for: Spatial distribution and habitat characterization of mosquito species during the dry season along the Mara River and its tributaries, in Kenya and Tanzania
Source: Infect Dis Poverty. 2018 Jan 18;7:2. doi: 10.1186/s40249-017-0385-0 (PMC5772712; doi:10.1186/s40249-017-0385-0)
Supplement: Additional file 1: — Multilingual abstracts in the six official working languages of the United Nations. (PDF 673 kb) [file 40249_2017_385_MOESM1_ESM.pdf]

Translation of the abstract into the six official working languages of the United Nations

التوزيع المكاني وخصائص مواطن معيشة فصائل حشرة البعوض خلال موسم الجفاف على طول نهر مارا وروافده، في كينيا وتنزانيا

غابرييل أو ديدا، دوغلاس ن. أنيونا، بول أو. أبوم، دانيال أكوكو، سامسون أو. أدوكا، ألي سعيد ماتانو، فيليب أو. أوار، كولينز أوما

ملخص

خلفية: لقد باتت مشكلة الأمراض المتناقلة بواسطة الحشرات تشكل مشكلة صحية كبيرة ومتزايدة في المجتمعات التي تعيش بجوار الأنهار الرئيسية في أفريقيا. وعلى الرغم من بحوث المسطحات المائية الكبيرة كالبحيرات والسدود التي تم إجراؤها على نطاق واسع، إلا أنه غالبا ما يتم اغفال الأنهار وروافدها إلى حد كبير. تهدف هذه الدراسة إلى تحديد التوزيع المكاني لفصائل حشرة البعوض خلال موسم الجفاف، بالإضافة إلى وصف مواطنها على طول نهر مارا وروافده.

الأساليب المستخدمة: تم في هذه الدراسة الاستعراضية أخذ عينات من يرقات البعوض المتواجدة على طول نهر مارا وروافديها الدائمين (رافد أمالا ورافد نيانغوريس)، والجداول النهرية الجافة، والمواطن المائية المجاورة (كالمستنقعات، والبرك التي تتعرض لأشعة الشمس المباشرة، والأحواض الصخرية ، و وحوافر حيوان فرس النهر و الماشية، والأحواض النباتية). وقد تم فحص كل موطن 20 مرة باستخدام مغرفة القياس المعيارية. تم تحديد المسافة بين مواقع تكاثر البعوض ومناطق الإقامة البشرية باستخدام إحداثيات نظام تحديد المواقع العالمي. تم تحديد يرقات البعوض التي تم جمعها باستخدام جهاز معياري لقياس أصناف الحيوانات. المياه تم قياس البارامترات الفيزيائية والكيميائية في الموقع باستخدام متر متعدد الأقطاب. تم مقارنة متوسط يرقات البعوض في كل المواطن على اختلاف أنواعها باستخدام تحليل التباين واختبارات مربع مربع، في حين تم تقييم العلاقة بين يرقات البعوض والبارامترات الفيزيائية والكيميائية باستخدام النموذج الخطي المعمم. تم استخدام اختبار كوكس-ستيوارت للكشف عن توجهات توزع يرقات البعوض. وقد مكّن هذا الاختبار من التحقق من التوجهات الرتيبة (أي رفض فرضية العدم المتعلقة بانعدام التوجه) وعامل التغير المرتبط بها.

النتائج: تم جمع ما مجموعه 4001 يرقة بعوض، منها 2712 (67.8٪) تم جمعها في المواطن النهرية / حافة الجداول و 1289 (32.2٪) عينة من المواطن المائية المتواجدة في النظام البيئي الأرضي على بعد 50 متر تقريبا من النهر / الجداول النهرية الرئيسية. تعد بعوضة الأنوفوليس غامبيا وبعوضة ارابينسيس وبعوضة فانيستيس ، أكثر ثلاث حشرات ناقلة لمرض الملاريا في جنوب الصحراء الكبرى في قارة أفريقيا، إلى جانب بعوض الأنوفلين الأخرى التي تشكل أكثر أنواع البعوض انتشارا بنسبة (70.3٪)، تليها بعوضة الكيولكس كوينكيفاسكياتس وبعوضة CX التي تبلغ نسبتها المشتركة (29.5٪). وقد تسببت الجداول النهرية المتجففة بتحقيق أكبر عدد ليرقات البعوض التي تم جمعها مقارنة بأنواع المواطن الأخرى. لوحظ وجود علاقة أقوى بين وفرة يرقات البعوض والأكسجين المذاب (Z = 7.37, P ≤ 0.001)، ، ودرجة الحرارة (= 7.65Z، P ≤ 0.001)، والتعكر (= 5.25-Z، P ≤ 0.001)، والمسافة إلى أقرب موطن بشري (= 4.57Z، P ≤ 0.001).

الاستنتاجات: يتطلب وجود يرقات البعوض الناقلة وغير الناقلة لمرض الملاريا داخل حوض نهر مارا اتخاذ إجراءات فورية للحد من تفشي الأمراض المتناقلة بواسطة الحشرات داخل الحوض. وينبغي تنفيذ برنامج لمكافحة الأمراض المتناقلة بواسطة البعوض خلال موسم الجفاف، واستهداف الجداول النهرية التي تتعرض للتجفيف التي قد تبين أنها تتسبب بوجود أعداد متزايدة ليرقات البعوض.

Translated from English version into Arabic by Nada Khwairah, through

TRANSLATORS
WITHOUT BORDERS

肯尼亚和坦桑尼亚境内马拉河及其支流蚊虫种类在旱季的空间分布和栖息地特征

Gabriel O Dida, Douglas N Anyona, Paul O Abuom, Daniel Akoko, Samson O Adoka, Ally-Said

Matano, Philip O Owuor, and Collins Ouma

摘要

引言：媒介传播疾病正日益成为非洲主要河流沿岸社区的主要健康问题。虽然湖泊和大坝等较大的水体已被广泛研究，但在很大程度上忽视了河流及其支流。本研究的目的是研究旱季蚊虫种类的空间分布，并进一步描述它们在马拉河及其支流的栖息地特征。

方法：本次横断面调查中，沿马拉河在其两大支流（Amala 和 Nyangores），即将干涸的小溪和邻近的水生栖息地（如沼泽、阳光直射的水洼、岩石坑、河马和牲畜的蹄印和植被池）采集蚊幼虫。每个栖息地用标准勺取样 20 次。利用全球定位系统坐标确定孳生地与人类居住地之间的距离。采用标准分类检索表鉴定蚊幼虫。用多参数测量仪原位测定水理化参数。用方差分析和卡方检验比较每种栖息地的平均蚊幼虫数，并用广义线性混合模型评估蚊幼虫与水理化参数之间的关系。运用 Cox-Stuart 检验测试蚊幼虫的分布趋势，该检验允许检测其单调趋势（拒绝 H0）及其变异性。

结果：共采集蚊幼虫 4001 只，其中，来自河流或小溪边缘栖息地的 2712 只（67.8%），1289 只（32.2%）采自距主要河流 50 m 的陆地生态系统的水生栖息地。在撒哈拉沙漠以南非洲地区，3 种最有效的疟疾传播媒介：冈比亚按蚊（*Anopheles gambiae* s.s.）、阿拉伯按蚊（*An. arabiensis*）和催命按蚊（*An. funestus* group），以及其他按蚊，均为优势蚊种（70.3%），其次是致倦库蚊（*Culex quinquefasciatus*）和尖音库蚊复合体（*Cx. pipiens* complex 29.5%）。与其他生境类型相比，即将干涸的溪流捕获的幼虫数量最多。蚊幼虫的丰度与溶解氧（Z = 7.37，$P \leqslant 0.001$）、温度（Z = 7.65，$P \leqslant 0.001$）、浊度（Z = -5.25，$P \leqslant 0.001$），以及距离最近的人类居住地（Z = 4.57，$P \leqslant 0.001$）之间有强烈的相关性。

结论：马拉河流域内有传疟蚊虫和非传疟蚊虫的幼虫，需立即采取行动以减少流域内媒传疾病的暴发。在旱季应该开展媒介控制工作，重点是孳生蚊幼虫数量最高的即将干涸的溪流。

Translated from English version into Chinese by Translated by Yin-Long Li, edited by Pin Yang

CHINESE CENTER FOR DISEASE CONTROL AND PREVENTION
NATIONAL INSTITUTE OF PARASITIC DISEASES

Répartition spatiale et caractérisation de l'habitat des espèces de moustiques pendant la saison sèche le long de la rivière Mara et de ses affluents, au Kenya et en Tanzanie

Gabriel O Dida, Douglas N Anyona, Paul O Abuom, Daniel Akoko, Samson O Adoka, Ally-Saïd Matano, Philip O Owuor et Collins Ouma

Résumé

Contexte: Les maladies à transmission vectorielle deviennent de plus en plus un problème de santé majeur pour les communautés vivant le long des principaux fleuves d'Afrique. Bien que les grandes étendues d'eau telles que les lacs et les digues ont fait l'objet de recherches approfondies, les cours d'eau et leurs affluents ont été largement ignorés. Cette étude visait à établir une répartition spatiale des espèces de moustiques pendant la saison sèche et à caractériser davantage leurs habitats le long de la rivière Mara et de ses affluents.

Méthodes: Dans cette étude transversale, des larves de moustiques ont été échantillonnées le long de la

rivière Mara, de ses deux affluents pérennes (Amala et Nyangores), des cours d'eau asséchés et des habitats aquatiques adjacents (marécages, flaques d'eau). , empreintes de sabots d'hippopotames et d'élevage, et piscines végétalisées). Chaque habitat a été plongé 20 fois à l'aide d'un plongeur standard. La distance entre les sites de reproduction et l'habitat humain a été déterminée en utilisant les coordonnées du système de positionnement global. Les larves de moustiques collectées ont été identifiées à l'aide de clés taxonomiques standard. Eau ples paramètres hysico-chimiques ont été mesurés sur place à l'aide d'un multimètre. Les larves moyennes de moustiques par type d'habitat ont été comparées en utilisant l'analyse de variance et les tests du chi carré, tandis que la relation entre les larves de moustiques et les paramètres physico-chimiques a été évaluée en utilisant un modèle mixte linéaire généralisé. Le test de Cox-Stuart a été utilisé pour détecter les tendances de la distribution des larves de moustiques. Le test a permis de vérifier la tendance monotone (rejet de l'hypothèse nulle d'absence de tendance) et sa variabilité.

Résultats: Un total de 4001 larves de moustiques ont été collectées, dont 2712 (67,8%) ont été collectées dans des habitats fluviaux et riverains et 1289 (32,2%) ont été échantillonnées dans des habitats aquatiques situés dans l'écosystème terrestre à environ 50 mètres de la rivière principale. . *Anopheles gambiae* ss, *Un. groupe d'arabiensis*, et *de. funestus* , les trois vecteurs les plus puissants du paludisme en Afrique subsaharienne, avec d'autres moustiques anophèleses, étaient les espèces de moustiques les plus dominantes (70,3%), suivies de *Culex quinquefasciatus* et *de Cx. pipiens* complexe combiné (29,5%). Les cours d'eau de séchage comptaient le plus grand nombre de larves capturées par rapport aux autres types d'habitat. Une relation plus forte entre l'abondance des larves de moustiques et l'oxygène dissous (Z = 7,37, $P \leq 0{,}001$), température (Z = 7,65, $P \leq 0{,}001$), turbidité (Z = -5,25, $P \leq 0{,}001$), et la distance à l'habitation humaine la plus proche (Z = 4,57, $P \leq 0{,}001$), a été observé.

Conclusions: La présence de paludisme et de larves de moustiques autres que le paludisme dans le bassin de la rivière Mara appelle à une action immédiate pour endiguer l'insurrection des maladies à transmission vectorielle dans le bassin. Un programme de lutte antivectorielle devrait être mené pendant la période sèche, en ciblant les cours d'eaux en dessiccation qui produisent le plus grand nombre de larves de moustiques.

Translated from English version into French by Abdourahamane, through

Пространственное распределение и характеристика мест обитания видов комаров в сухой сезон в бассейне реки Мара и ее притоков в Кении и Танзании

Габриэль О Дида, Дуглас Н Аньона, Пол О Абуом, Даниэль Акоко, Самсон О Адока, Алли-Саид Матано, Филип Ouoop и Коллинз Ума

Аннотация

Обоснование исследования. Трансмиссивные болезни все чаще становятся серьезной

проблемой для здоровья людей, живущих вдоль крупных рек Африки. Если большие водоемы, такие как озера и огороженные плотинами резервуары, были тщательно обследованы, то реки и их притоки в основном не были изучены. Это исследование было направлено на выявление пространственного распределения видов комаров в течение сухого сезона и дальнейшее определение характеристик их мест обитания в бассейне реки Мара и ее притоков.

Методы. В этом межгрупповом исследовании были взяты образцы личинок комаров вдоль реки Мара, ее двух постоянных притоков (Амала и Ньянгорес), пересыхающих потоков и прилегающих водных сред обитания (таких как, болота, получающие прямой солнечный свет лужи [открытые, освещенные солнцем лужи], скопления воды среди камней, вмятины от копыт бегемотов и домашнего скота, заросшие водоемы). В каждой среде обитания было взято 20 проб с использованеим стандартного ковша. Расстояние между местами размножения и жильем людей определялось при помощи координат глобальной системы позиционирования. Собранные личинки комаров были идентифицированы с использованием стандартных таксономических ключей. Физико-химические показатели воды измерялись на месте при помощи многопараметрического счетчика. Сравнивалось среднее количество личинок комаров на тип среды обитания с применением анализа дисперсии и критериев хи-квадрат, и связь между личинками комаров и физико-химическими показателями оценивалась с использованием обобщенной линейной смешанной модели. Критерий Кокса-Стюарта использовался для выявления тенденций распределения личинок комаров. Этот критерий позволил проверить монотонную тенденцию (опровержение нулевой гипотезы об отсутствии тренда) и ее изменчивость.

Результаты. Было собрано в общей сложности 4001 личинок комаров, из которых 2712 (67,8%) были получены в местах обитания по берегам рек/потоков и 1289 (32,2%) были отобраны в водных местах обитания, расположенных в наземной экосистеме примерно в 50 метрах от основных рек/потоков. *Anopheles gambiae* s.s., *An. arabiensis*и *An. funestus,* три наиболее потентные группы носителей малярии в Африке к югу от Сахары, вместе с другими малярийнымикомарами, являются самыми доминирующими видами (70,3%), за которыми следуют *Culex quinquefasciatus* и *Cx. pipiens* совместно (29,5%). В пересыхающих потоках было насчитано наибольшее количество личинок по сравнению с другими типами мест обитания. Была замечена более сильная связь между обилием личинок комаров и растворенным кислородом ($Z = 7,37$; $P \leq 0,001$), температурой ($Z = 7,65$; $P \leq 0,001$), мутностью ($Z = -5,25$; $P \leq 0,001$) и расстоянием до ближайшего человеческого жилья ($Z = 4,57$; $P \leq 0,001$).

Выводы. Наличие личинок малярийных и немалярийных комаров в бассейне реки Мара требует немедленных действий по предупреждению резкого увеличения трансмиссивных заболеваний в этой районе. Борьба с переносчиками болезней должна проводиться в течение сухого периода и в основном направлена на обработку пересыхающих потоков, в которых, как показало исследование, присутствует наибольшее количество личинок комаров.

Translated from English version into Russian by Natalia Potashnik, through

TRANSLATORS
WITHOUT BORDERS

Distribución espacial y caracterización del hábitat de especies de mosquitos durante la estación seca a lo largo del río Mara y sus afluentes, en Kenia y Tanzania

Gabriel O Dida, Douglas N Anyona, Paul O Abuom, Daniel Akoko, Samson O Adoka, Ally-Said Matano, Philip O Owuor y Collins Ouma

Resumen

Introducción. Las enfermedades transmitidas por vectores se están convirtiendo en un problema de salud cada vez más importante entre las comunidades que viven a lo largo de los principales ríos de África. Aunque las grandes masas de agua, como lagos y presas, han sido objeto de extensas investigaciones, los ríos y sus afluentes han sido en gran medida ignorados. El objetivo de este estudio fue establecer la distribución espacial de las especies de mosquitos durante la estación seca, así como caracterizar sus hábitats a lo largo del río Mara y sus afluentes.

Métodos. En este estudio transversal, se tomaron muestras de larvas de mosquitos a lo largo del río Mara, sus dos afluentes perennes (el río Amala y el río Nyangores), arroyos de caudal menguante y los hábitats acuáticos adyacentes (por ejemplo, pantanos, charcas que recibían luz solar directa [charcas abiertas y soleadas], pozas de marea, huellas de hipopótamo y de ganado, así como charcas con vegetación). De cada hábitat se tomaron muestras sumergiendo 20 veces usando un dipper (cucharón) estándar. Se determinó la distancia entre los sitios de cría y los asentamientos humanos utilizando las coordenadas del sistema de posicionamiento global (GPS). Las larvas de mosquito recolectadas se identificaron utilizando claves taxonómicas estándar. Los parámetros físico-químicos se midieron in situ con un medidor multiparamétrico. Se comparó el número medio de larvas de mosquitos por tipo de hábitat mediante análisis de varianza y pruebas de ji al cuadrado, mientras que la relación entre las larvas de mosquito y los parámetros físico-químicos se evaluó mediante un modelo lineal generalizado mixto. Se empleó el test de Cox-Stuart para detectar tendencias en la distribución de las larvas de mosquito. El test permitió la verificación de la tendencia monotónica (rechazo de la hipótesis nula de ausencia de tendencia) y su variabilidad.

Resultados. Se recolectó un total de 4001 larvas de mosquitos, de las cuales 2712 (67,8 %) fueron extraídas de los ecotonos de ríos y arroyos y 1289 (32.2%) fueron tomadas como muestras de hábitats acuáticos ubicados en el ecosistema terrestre a unos 50 metros de los ríos y arroyos principales. *Anopheles gambiae* s.s., *An. arabiensis* y el grupo del *An. funestus*, los tres vectores más potentes de la malaria en el África Subsahariana, junto con otros mosquitos anofelinos, fueron las especies de mosquito más dominantes (70,3 %), seguidas por *Culex quinquefasciatus* y el complejo *Cx. pipiens* combinados (29,5 %). De todos los tipos de hábitat, los arroyos de caudal menguante fueron el hábitat en el que se capturó el mayor número de larvas. Se observó una relación más fuerte entre la abundancia de larvas de mosquitos y el oxígeno disuelto ($Z = 7{,}37$, $P \leq 0{,}001$), temperatura ($Z = 7{,}65$, $P \leq 0{,}001$), turbidez ($Z = -5{,}25$, $P \leq 0{,}001$) y la distancia al asentamiento humano más cercano ($Z = 4{,}57$, $P \leq 0{,}001$).

Conclusiones. La presencia de larvas de mosquitos palúdicos y no palúdicos dentro de la cuenca del río Mara requiere medidas inmediatas para reducir la aparición de enfermedades transmitidas por vectores dentro de la cuenca. Se debería llevar a cabo un programa de control de vectores durante la estación seca, centrado en los arroyos de caudal menguante que hayan demostrado que producen el

mayor número de mosquitos larvales.

Translated from English version into Spanish by David Fuertes Severo, through

TRANSLATORS
WITHOUT BORDERS
